# Clinical, morphological, and molecular characterization of patients with X-linked myopathy with excessive autophagy (XMEA)

**DOI:** 10.1093/jnen/nlaf134

**Published:** 2025-11-27

**Authors:** Angèle N Merlet, Emmanuelle Lacène, Isabelle Nelson, Guy Brochier, Clémence Labasse, Anais Chanut, Angeline Madelaine, Maud Beuvin, Gisèle Bonne, Léonard Féasson, Marie-Christine Minot, Jean-Baptiste Noury, Mélanie Fradin, Marco Savarese, Gorka Fernández-Eulate, Anthony Behin, Tanya Stojkovic, Andreas Hentschel, Pascale Marcorelles, Andreas Roos, Teresinha Evangelista

**Affiliations:** Myology Unit, Reference Center for Neuromuscular Diseases, Euro-NMD, Department of Clinical Physiology and Exercice, University Hospital of Saint-Etienne, Saint-Priest-en-Jarez, France; Interuniversity Laboratory of Human Movement Biology, Jean-Monnet University, Saint-Etienne, France; Functional Unit of Neuromuscular Pathology, Department of Neuropathology, Institut de Myologie, GHU Pitié-Salpêtrière, Paris, France; Sorbonne University, Inserm, Institut de Myologie, Centre de Recherche en Myologie, Paris, France; Functional Unit of Neuromuscular Pathology, Department of Neuropathology, Institut de Myologie, GHU Pitié-Salpêtrière, Paris, France; Functional Unit of Neuromuscular Pathology, Department of Neuropathology, Institut de Myologie, GHU Pitié-Salpêtrière, Paris, France; Functional Unit of Neuromuscular Pathology, Department of Neuropathology, Institut de Myologie, GHU Pitié-Salpêtrière, Paris, France; Functional Unit of Neuromuscular Pathology, Department of Neuropathology, Institut de Myologie, GHU Pitié-Salpêtrière, Paris, France; Functional Unit of Neuromuscular Pathology, Department of Neuropathology, Institut de Myologie, GHU Pitié-Salpêtrière, Paris, France; Sorbonne University, Inserm, Institut de Myologie, Centre de Recherche en Myologie, Paris, France; Myology Unit, Reference Center for Neuromuscular Diseases, Euro-NMD, Department of Clinical Physiology and Exercice, University Hospital of Saint-Etienne, Saint-Priest-en-Jarez, France; Interuniversity Laboratory of Human Movement Biology, Jean-Monnet University, Saint-Etienne, France; Neuromuscular Competence Center, University Hospital of Rennes, Rennes, France; Reference Centre for Neuromuscular Diseases AOC, University Hospital of Brest, Brest, France; Department of Medical Genetics, Hôpital Sud, University Hospital of Rennes, Rennes, France; Folkhälsan Research Center, Helsinki, Finland; AP-HP, Reference Center for Neuromuscular Disorders, Institut de Myologie, Hôpital Pitié-Salpêtrière, Paris, France; AP-HP, Reference Center for Neuromuscular Disorders, Institut de Myologie, Hôpital Pitié-Salpêtrière, Paris, France; AP-HP, Reference Center for Neuromuscular Disorders, Institut de Myologie, Hôpital Pitié-Salpêtrière, Paris, France; Leibniz-Institut für Analytische Wissenschaften—ISAS—e. V, Dortmund, Germany; Department of Pathology, University Hospital of Brest, Brest, France; Department of Pediatric Neurology, Centre for Neuromuscular Disorders, Centre for Translational Neuro- and Behavioral Sciences, University Hospital Essen, University Duisburg-Essen, Essen, Germany; Functional Unit of Neuromuscular Pathology, Department of Neuropathology, Institut de Myologie, GHU Pitié-Salpêtrière, Paris, France

**Keywords:** autophagic vacuolar myopathy, skeletal muscle, ultrastructure, VMA21, proteomic, X-linked myopathy with excessive autophagy, XMEA

## Abstract

X-linked myopathy with excessive autophagy (XMEA) is a slowly progressive disease affecting male patients, caused by hemizygous mutations in the *VMA21* gene. We studied nine patients from six unrelated French families clinically suspected of having XMEA. Clinical charts were reviewed, and muscle biopsies underwent histological, immunohistochemical, and electron microscopy analysis. Sanger sequencing and next generation *VMA21* gene panels were performed, and proteomic profiling was done on muscle extracts from two patients. Clinical onset ranged from childhood to adulthood with most showing proximal lower limb weakness and mild creatine kinase elevation. Three patients had cardiac and respiratory involvement. Muscle biopsies revealed cytoplasmic vacuoles, split fibers, internalized nuclei and variable fiber sizes. Vacuoles stained positively for sarcolemmal and autophagic proteins, as well as for complement C5b-9. Ultrastructural analysis showed basal lamina duplication, subsarcolemmal vacuoles, and extensive autophagosome extrusion. Proteomic analysis revealed complement activation, impaired proteolysis, and mitochondrial/cytoskeletal vulnerabilities. Biglycan and thrombospondin-4 were identified as potential novel diagnostic markers. Molecular studies found two known pathogenic variants (c.164-7T>G and c.163 + 4A>G) and a novel 3′UTR variant (c.*124A>G) in *VMA21*. This study expands the clinical spectrum of XMEA by reporting adult-onset cases, a novel mutation, and highlights the value of proteomics in understanding the pathophysiology of XMEA.

## INTRODUCTION

Inherited myopathies linked to defective lysosomal function are increasingly recognized; ([XMEA], OMIM 310440) represents a prototypical example. XMEA is a rare neuromuscular disorder first described in 1988, with onset typically occurring during childhood.^1^ To date, all affected individuals have been male. The disease is characterized by progressive proximal muscle weakness, initially affecting the lower limbs and subsequently spreading to other muscle groups, ultimately leading to loss of ambulation around the age of 50 years.^1^^,^^2^ Other organs are generally spared, although respiratory insufficiency is relatively common, while cardiac involvement remains rare.^3^ Nevertheless, rare cases of extramuscular involvement have been reported, including cardiac abnormalities and liver dysfunction, notably associated with congenital disorders of glycosylation presenting with autophagic liver disease.^4–7^ Overall, XMEA is considered a mild, slowly progressive condition, although considerable clinical variability exists;^3^ this includes late-onset forms^8^ and more severe presentations, either congenital^4^^,^^7^ or emerging during early adulthood.^9^

XMEA is caused by mutations in the *VMA21* gene located on Xq28.^10^  *VMA21* encodes the vacuolar ATPase assembly factor (VMA21), a critical chaperone required for the assembly of the principal mammalian proton pump involved in lysosomal acidification. Loss of VMA21 function leads to the accumulation of autophagic vacuoles with sarcolemmal features.^11^ Histopathological analyses of skeletal muscle biopsies reveal an excessive number of cytoplasmic vacuoles, deposition of complement of membrane attack complex (C5b-9) within vacuoles and along the sarcolemma,^12^ and, in some cases, aggregates of a classical receptor of autophagy (p62) and TAR DNA-binding protein 43 (TDP-43).^3^ Electron microscopy demonstrates subsarcolemmal and intermyofibrillar vacuoles, occasional vacuoles abutting the muscle fiber membrane, abundant membrane-bound clustered lysosomes, and thickening or duplication of the basal lamina in abnormal fibers.^12^

Mutations in *VMA21* impair vacuolar ATPase assembly, resulting in decreased enzymatic activity, defective lysosomal acidification, and blockade of autophagy.^12^ Pathogenic variants are primarily located within intronic regions, affecting splicing branch points, donor sites, or acceptor sites.^3^^,^^10^ Additionally, two exonic mutations impacting splice enhancer elements have been reported,^3^^,^^4^^,^^10^^,^^13^^,^^14^ as well as a mutation located at the beginning of the 3' untranslated region.^3^^,^^10^^,^^15^^,^^16^ All of these variants lead to a reduction or loss of VMA21 protein abundance.^6^^,^^8^^,^^10^^,^^17^ There appears to be a genotype-phenotype continuum, depending on the extent to which protein expression or function is compromised, influencing disease severity.^18^ However, the precise mechanisms underlying some mutations remain unclear. Recent studies demonstrated that the VMA21-120 isoform is predominantly expressed in skeletal muscle and may be a key contributor to XMEA pathogenesis.^19^ Moreover, XMEA-associated mutations lead to the deficiency of both VMA21-101 and VMA21-120 isoforms.^19^

In this multicenter retrospective study, we present the clinical, histopathological, and proteomic features of a large French cohort of nine patients with genetically confirmed XMEA. Our proteomic analysis reveals novel diagnostic-relevant protein signatures and provides new insights into XMEA pathophysiology, considering the heterogeneity of disease severity.

## METHODS

### Patients

Data from the patients originated from the Pitié-Salpêtrière Hospital in Paris and two other neuromuscular reference centers in France (Brest and Rennes). In nine patients from six unrelated French families, the clinical presentation was suggestive of XMEA (Table 1). Two patients from one family have been described in a previous study,^20^ while patients from the other five families represent newly identified cases. Clinical data were gathered through a comprehensive review of all available medical records up to the most recent clinical examination. The following parameters were considered: age at disease onset (defined as the first reported disease-related symptom, including delayed motor milestones), pattern of muscle weakness (proximal and distal limb muscles, facial muscles), presence of ptosis, external ophthalmoplegia, dysphagia, contractures, tendon reflexes, respiratory and cardiac involvement, serum creatine kinase levels, and electromyography (EMG) findings. The severity of the clinical phenotype was also assessed and categorized as follows: mild (late-adult onset or mild muscle weakness), or severe (childhood onset, severe muscle weakness, or presence of cardiac/respiratory involvement). All clinical evaluations were conducted by experienced neuromuscular specialists with over 30 years of expertise in the field.

**Table 1.** Detailed clinical features of the cohort.

	Family 1		Family 2	Family 3	Family 4	Family 5		Family 6	
Patients	*a*	*b*	*c*	*d*	*e*	*f*	*g*	*h*	*i*
**Sex**	Male	Male	Male	Male	Male	Male	Male	Male	Male
**Age at clinical examination**	50	64	24	35	51	17	53	9	65
**Age of onset**	Childhood	Adulthood	Childhood	Childhood	Adulthood	Childhood	Adulthood	Childhood	Adulthood
**Walking aid, years**	48 (cane)	55 (cane) 56 (crutches)	?	35 (cane) 48 (wheelchair)	No	No	?	No	No
**Mutation**	c.164-7T>G	c.164-7T>G	c.164-7T>G	c.163 + 4A>G	c.124A>G	c.164-7T>G	c.164-7T>G	c.164-7T>G	c.164-7T>G
**Clinical severity**	Moderate	Moderate	Severe	Severe	Mild	Moderate	Mild	Severe	Mild
**Clinical manifestations**									
Proximal limb weakness	Yes (LL: +++; UL: ++)	Yes (LL: +++; UL: ++)	Yes (LL: ++; UL: +)	Yes (LL: +++, UL: ++)	No	Yes (LL : +, UL −)	Yes (LL:+++, UL: +)	No	Yes (LL : ++)
Distal limb weakness	Yes (LL: −; UL: +++)	Yes (LL: ++; UL: +)	No	Yes (LL: ++, UL: NA)	No	No	Yes (LL: ++, UL: +)	No	No
Axial weakness	Yes (++)	Yes (+)	Yes (++)	Yes (++)	No	No	No	No	No
Ptosis	No	No	No	No	No	No	No	No	No
External ophthalmoplegia	No	No	No	No	No	No	No	No	No
Facial weakness	No	No	No	No	No	No	No	No	No
Dysphagia	No	No	No	No	No	No	No	No	No
Respiratory involvement	Yes	No	No	Yes	No	No	No	No	Yes
Cardiac involvement	No	No	No	Yes	No	No	Yes	No	Yes
**Tendon reflexes**	No	Yes	Yes	Yes	Yes	Yes	–	Yes	NA
**Contractures**	No	No	No	Yes	No	Yes	–	No	No
**Creatine kinase, IU/L**	240	121	913	686	890	1095	450	1717	380
**Electromyography**	Myogenic pattern with pseudo-myotonic discharge	Myogenic pattern	Myogenic pattern with pseudo-myotonic discharge	Myogenic pattern with pseudo-myotonic discharge	Myogenic pattern	Myogenic pattern	Myogenic pattern	Myogenic pattern	NA

LL, lower limbs; UL, upper limbs; NA, not available. Kendall’s Muscle Testing: ≤ 2 (+++); < 3–4 < (++); = 4 (+).

### Genetic analysis

Deoxyribonucleic acid (DNA) derived from the XMEA patients was obtained from the Genethon laboratory (Evry) and was extracted from EDTA-blood samples. The reference sequence of *VMA21* is ENST00000330374.7-M_001017980.4. Primers were selected using Primer3 software (https://primer3.ut.ee) and synthesized by Eurogentec (http://www.eurogentec.com) (Table 2). After optimization of specific band amplification conditions, polymerase chain reaction (PCR) products were purified on column (NucleoSpin- Gel and PCR clean-up- Macherey Nagel) and Sanger sequencing was performed by Eurofins Genomics. The search for variants in the sequences obtained is carried out by Blast (BLAST ^®^ “ blastn suite) on the reference sequence.

**Table 2.** Histological features of the cohort.

	Family 1		Family 2	Family 3	Family 4	Family 5		Family 6	
Patients	*a*	*b*	*c*	*d*	*e*	*f*	*g*	*h*	*i*
**Age at biopsy, years old**	50	64	24	37	51	17	34	11	62
**Muscle**	Deltoid	Deltoid	Deltoid	Deltoid	Radial	Quadriceps	NA	Quadriceps	Biceps
**Fiber size variability**	Yes (+++)	Yes (+++)	Yes (+)	Yes (++)	Yes (+)	Yes (++)	Yes (+++)	Yes (+)	Yes (++)
**Atrophic fiber**	Yes (+++)	Yes (+++)	Yes (+)	Yes	Yes (++)	Yes (++)	Yes (+++)	Yes (+)	Yes (+++)
**Group of atrophic fibers**	Yes (+++)	Yes (+++)	No	Yes (+)	Yes (+)	Yes (+)	Yes (+++)	No	Yes (++)
**Hypertrophic fiber**	Yes (+++)	Yes (+++)	Yes (+)	Yes (+)	Yes (++)	Yes (++)	Yes (++)	Yes (+)	Yes (+)
**Fiber shape**	Rounded	Rounded	Rounded	Rounded	Polygonal	Rounded	Polygonal	Polygonal	Polygonal
**Splitted fiber**	Yes (+++)	Yes (+++)	Yes (++)	Yes (++)	Yes (+)	Yes (+)	Yes (+++)	Yes (+)	Yes (++)
**Internalized nuclei, % of fiber**	Yes (+++, 28%)	Yes (+++, 29%)	Yes (+++, 34%)	Yes (+, 6%)	Yes (+, 7%)	Yes (+, 10%)	Yes (+++, 24%)	Yes (+, 4%)	Yes (+++, 29%)
**Vacuoles, % of fiber**	Yes (+++, 36%)	Yes (+++, 25%)	Yes (+++, 25%)	Yes (++, 18%)	Yes (+++, 30%)	Yes (+++, 37%)	Yes (+++, 58%)	Yes (+++, 21%)	Yes (+, 4%)
**Increased connective tissue**	Yes (+++)	Yes (+)	Yes (+)	Yes (++)	Yes (+)	Yes (++)	Yes (++)	No	Yes (+)
**Necrosis**	No	Yes (+)	Yes (+)	No	No	No	No	No	No
**Nuclear bags**	Yes (++)	Yes (+)	Yes (+)	Yes (+)	No	Yes (++)	Yes (+)	No	Yes (+++)
**Muscle cell membrane with a granular or dotted aspect**	Yes (++)	Yes (+)	Yes (+)	Yes (++)	No	No	Yes (+)	Yes (+)	Yes (+)
**Ragged red fibers**	Yes (+)	Yes (++)	No	Yes (+)	No	No	No	No	No
**Mitochondrial accumulations in the periphery of the fibers**	Yes	Yes	No	No	Yes	No	No	No	Yes (+)
**Myofibrillar network disorganisation**	No	No	No	Yes	No	No	No	No	No
**Predominance of a fiber type**	No	Yes (type I)	No	Yes (type I)	No	No	No	No	Yes (type I)
**Preferential atrophy of a fiber type**	Yes (type II)	Yes (type II)	Yes (type II)	Yes (type II)	Yes (type I)	No	No	No	No

Morphometric alterations quantified as follows: few (+), mild (++) or severe (+++) alterations. Quantitative analysis of internalized nuclei and vacuoles was performed on the entire muscle cross-section as a percentage of the number of fibers.

### Histological studies on muscle biopsy specimens

Open muscle biopsies were obtained from various muscles (deltoid, *n* = 4; radial, *n* = 1; biceps, *n* = 1; quadriceps, *n* = 2). For one patient, the biopsy site is unknown. The diverse origins of the muscle biopsies offer a valuable opportunity to study XMEA-associated characteristics, including biochemical features, across different muscles, which in turn enabled us to identify common biochemical key players with diagnostic relevance in terms of tissue markers. Histological and histochemical slides were systematically re-analyzed by two authors (A.N.M. and T.E.), both with experience in skeletal muscle morphology and pathology. Conventional histological and histochemical techniques were performed on 8–10-μm-thick cryostat sections. These included hematoxylin and eosin (H&E), modified Gomori trichrome (GT), Periodic acid–Schiff (PAS), Oil Red O, reduced nicotinamide adenine dinucleotide dehydrogenase-tetrazolium reductase (NADH-TR), succinic dehydrogenase (SDH), cytochrome c oxidase (COX), adenosine triphosphatase (ATPase) preincubated at pH 9.4, 4.63, 4.35, and acid phosphatase stains. Digital photographs of biopsies were captured using a Zeiss epifluorescence microscope (Axioimager M2 Zeiss, Carl Zeiss GmbH, Germany). Manual quantification of fibers with internalized nuclei and vacuoles was performed on the entire muscle section by the same experimenter (A.N.M.). Other morphological variables were also considered: fiber size variability, atrophic fibers, groups of atrophic fibers, hypertrophic fibers, internalized nuclei, fibers with vacuoles, split fibers, increased connective tissue, nuclear bags, muscle cell membrane with a granular or dotted aspect, ragged red fibers, and mitochondrial accumulations in the periphery of the fibers. These morphological features were quantified as follows: low (+), mild (++), or severe (+++) alterations.

### Electron microscopy

Ultrastructural analysis was performed on all muscle biopsies. Small muscle specimens were fixed with glutaraldehyde (2.5%, pH 7.4), post fixed with osmium tetroxide (2%), dehydrated and embedded in resin. Longitudinally oriented ultra-thin sections were obtained at different level of depth from 1 to 3 small blocks and stained with uranyl acetate and lead citrate. Ultra-thin sections of transversally oriented blocks were obtained only for the most significant findings. The grids were observed using a JEM-1400FLASH electron microscope (80 kV; Philips Electronics NV, Eindhoven, The Netherlands) and were photo documented using a Xarosa camera (EMSIS GmbH, Münster, Germany).

### Proteomic profiling

To identify proteins affected by XMEA in different muscles, proteomic profiling was performed making use of a data-independent-acquisition approach on proteins extracts of muscle biopsies derived from only two patients, (due to the availability of tissue samples), and matching controls. To this end, we made use of a protocol published previously.^21^

### Immunohistochemistry and immunofluorescence

Immunohistochemistry and immunofluorescence analyses were performed in 6 of 9 patients from available frozen muscle samples. Antibodies against dystrophin/DMD (D8168, Sigma, St Louis, MO, USA), LC3 (L7543, Sigma), lysosomal-associated membrane protein (LAMP2) (ab25223, Abcam, Cambridge, UK), p62 (BD Biosciences, 610833, USA), C5b-9 (DakoCytomation, M 0777, Glostrup, Denmark), and TDP43 (10782-2-AP, Proteintech, Manchester, UK) were visualized using immunoperoxidase techniques. Antibodies against hnRNPA1 (D21H11, Cell Signaling Technology, Danvers, MA, USA), hnRNPA2/B1 (EF-67, Santa Cruz Biotechnology, Dallas, TX, USA), hnRNPA2/B1 (DP3B3, Santa Cruz Biotechnology), and valosin-containing protein (VCP) (MA3-004, Thermo Fisher Scientific, Waltham, MA, USA) were used and abundance and distribution of respective antigens were visualized using immunofluorescence techniques. For both techniques, primary antibodies were incubated overnight at 4°C and sections were subsequently incubated with appropriate conjugated secondary antibodies (Alexa Fluor-488 goat anti-rabbit antibody and Jackson IR goat anti-mouse antibody) for one hour. A set of control slides was prepared with the omission of the primary antibodies.

Verification of paradigmatic findings of the overall proteomic data by immunofluorescence analyses were performed in the two patients mentioned above and compared with one GNE myopathy patient (classical histopathology feature: rimmed vacuoles), and one control subject. The following primary antibodies were used: anti-thrombospondin-4 (THBS4) (1:100, PA5-68467, Thermo Fisher, Darmstadt, Germany) and anti-biglycan (1:100, PA5-76821, Thermo Fisher).

## RESULTS

Patients *a* and *b* of the family 1 were previously reported^20^ but are repeated here to extend and compare the clinical manifestations (Table 1) and the histopathology (Tables 2 and 3).

**Table 3.** Ultrastructural features of the cohort.

	Family 1		Family 2	Family 3	Family 4	Family 5
Patients	*a*	*b*	*c*	*d*	*e*	*f*
Replication of the basal lamina	Yes	Yes	Yes	Yes	Yes	Yes
Vacuoles in the extracellular matrix	Yes	–	Yes	Yes	Yes	Yes
Sub-sarcolemmal vacuoles	Yes	Yes	Yes	Yes	Yes	Yes
Cytoplasmic vacuoles	Yes	Yes	Yes	Yes	Yes	Yes
Vacuoles containing calcium	Yes	Yes	Yes	–	–	–
Proximity between nucleus and vacuole	Yes	Yes	Yes	–	Yes	Yes
Disorganization of the network	No	Yes	No	No	Yes	No
Clustering of nuclei	No	No	No	No	Yes	
Accumulation of collagen	Yes	Yes	Yes	Yes	Yes	Yes
Paracrystalline inclusion in the mitochondria	Yes	No	No	No	No	–

### Clinical presentation of the patients

Table 1 summarizes the clinical manifestations of nine patients from six unrelated French families. All patients are male. In five of the nine patients (*a*, *c*, *d*, *f*, and *h*), symptoms first appeared during childhood, (eg difficulty running or climbing a rope), and became more clinically apparent in early adulthood (eg difficulty climbing stairs). Some patients (*a*, *b*, and *d*) eventually required a cane for walking, while patient *d* progressed to using a wheelchair. Patients *f* and *h*, aged 17 and 9 respectively, are still ambulatory without assistance. In the remaining four patients (*b*, *e*, *g*, and *i*), symptoms began in early adulthood. Among them, only patient *b* required a cane to walk. Muscle weakness in all patients predominantly affected the pelvic-femoral region, although muscle strength remained intact in the youngest patient (*h*) and in patient *e*. In some patients, weakness extended to the proximal upper limbs (*a*, *b*, *c*, *d*, and *g*), distal lower limbs (*b*, *d*, and *g*), and distal upper limbs (*a*, *b*, and *g*). None of the patients showed signs of ptosis, external ophthalmoplegia, facial weakness, or dysphagia. Cardiac involvement was noted in three patients. Patient *d* developed rhythm disorders requiring pacemaker implantation at age 43. Patient *g* had a left anterior hemiblock on ECG, and patient *i* underwent aortic valve surgery. Restrictive respiratory insufficiency was reported in three patients. Vital capacity was 31% in patient *d* and 50% in patient *a*. Although the exact value was unknown for patient *i*, he required ventilatory support. Tendon reflexes were preserved in eight of the nine patients, and two patients (*d* and *f*) exhibited joint contractures. Creatine kinase levels were mildly to moderately elevated in eight patients, ranging from 240 to 1717 U/L. EMG revealed a myogenic pattern in eight patients, with pseudo-myotonic discharges observed in some cases.

### Genetic analysis

Sanger sequencing or next-generation panel-based sequence analysis of *VMA21* revealed a known pathogenic splice site mutation, upstream of the third exon (c.164-7T>G), in five patients from three different families (family 2: *c*; family 5: *f*, the grandson of *g*; family 6: *h*, the half great-uncle of *i*). This is the same mutation already reported in patients *a* and *b* of family 1 (first cousins).^10^ We also report another known splice site mutation, previously identified in French and Brazilian families,^10^^,^^14^ downstream of the second exon (c.163 + 4A>G) in patient *d*. Additionally, we identified a novel variant in the 3'UTR region of the gene in patient *e* (c.124A>G).

### Histological and immunohistochemical analysis

**Figure 1.**
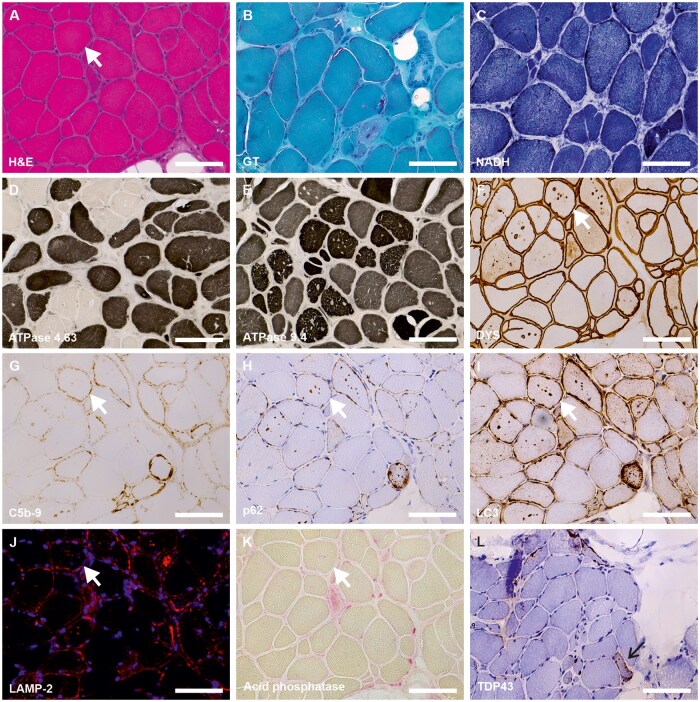
Histopathological findings in muscle biopsy from a patient with XMEA. Light microscopy analysis demonstrated: (A) marked fiber size variability, numerous fibers containing multiple cytoplasmic vacuoles, internalized nuclei, fiber splitting, and increased endomysial connective tissue; (B) a granular or dotted aspect of the muscle cell membrane and red labeling of cytoplasmic vacuoles; (C) absence of myofibrillar disorganization but peripheral accumulations of mitochondria in some fibers; (D, E) predominance of type I fibers and selective atrophy of type II fibers; (F) dystrophin labeling on vacuoles and muscle fiber membranes; (G) deposition of complement C5b-9 within vacuoles and along muscle fiber membranes; (H–J) positive labeling of p62, LC3, and LAMP-2 (red) on autophagic vacuoles and membranes of abnormal fibers (nuclei counterstained in blue); (K) weak acid phosphatase activity within vacuoles; (L) cytoplasmic TDP-43 aggregates in some muscle fibers (black arrow). The same abnormal fiber is indicated by the white arrow across serial sections. Scale bar = 100 µm for all panels. Images are from patient *d*. Abbreviations: C5b-9, complement membrane attack complex; DYS, dystrophin; GT, Gömöri trichrome; H&E, hematoxylin and eosin; LAMP-2, lysosome-associated membrane protein 2; LC3, autophagy marker light chain 3; NADH-TR, reduced nicotinamide adenine dinucleotide dehydrogenase-tetrazolium reductase.

Morphological analyses are presented in Table 2. Overall, the histological features of the muscle tissue were similar among patients. Figure 1 illustrates the muscle biopsy of patient *d*. Muscle biopsy images from the remaining patients are presented in Supplementary Figure 1 to 8. H&E staining revealed numerous vacuoles in the muscle fibers in eight of the nine patients (18–58% of fibers), often multiple within a single fiber and appearing optically empty (Figure 1A). Only patient *i* displayed fewer vacuoles (4% of muscle fibers) compared to the others. All patients exhibited a high number of internalized myonuclei (4–34% of fibers), often multiple within a single fiber, along with numerous split fibers (Figure 1A). Fiber size variation was marked in four patients, particularly in the older ones, with both hypertrophic and atrophic fibers observed. This was associated with increased endomysial connective tissue in eight of the nine patients. Groups of atrophic fibers were noted in seven patients. Fiber shape was rounded in five patients and polygonal in the remaining four. Nuclear bags were identified in seven of the nine patients. Necrosis was reported in only two cases. GT staining revealed a granular or dotted aspect of the muscle cell membrane in abnormal cells (seven of nine patients), mitochondrial accumulations at the periphery of fibers (three patients), and ragged-red fibers in three patients (Figure 1B). NADH-TR staining did not show major disorganization of the myofibrillar network (Figure 1C), though slight disorganization was noted in muscle biopsies from two patients. ATPase staining revealed a predominance of type I fibers in three patients and preferential atrophy of type II fibers in four of eight patients (Figure 1D, E). PAS and Oil Red O (or Sudan Black) staining did not indicate glycogen or triglyceride accumulation in vacuoles (data not shown).

Muscle protein analysis was performed in six of the nine patients (*d* to *i*) using immunohistochemistry and immunofluorescence on available muscle samples. Findings were consistent across patients. In the selected specimens, dystrophin antibodies labeled both the sarcolemma and cytoplasmic vacuoles (Figure 1F). C5b-9 antibody strongly labeled the sarcolemma of abnormal fibers and certain vacuoles (Figure 1G). Autophagy-related markers p62 and LAMP-2 showed strong labeling of multiple cytoplasmic vacuoles and the periphery of abnormal fibers (Figure 1H, J). Acid phosphatase activity was detected in vacuoles but was relatively low in intensity (Figure 1K). In two of the six patients (*d* and *e*), additional staining for LC3 and TDP-43 was performed.

LC3 labeling was strong in cytoplasmic vacuoles and at the periphery of abnormal fibers, similar to LAMP-2 (Figure 1I). Slight cytoplasmic aggregates were observed in a few fibers with the TDP-43 antibody (Figure 1L). In these two patients, labeling for heterogeneous nuclear ribonucleoproteins (hnRNPA1 and hnRNPA2/B1) was also performed to exclude multisystem proteinopathies. No cytoplasmic aggregates were detected (data not shown). Similarly, no VCP-immunoreactive cytoplasmic aggregates were observed in any muscle fibers (data not shown).

### Electron microscopy

**Figure 2.**
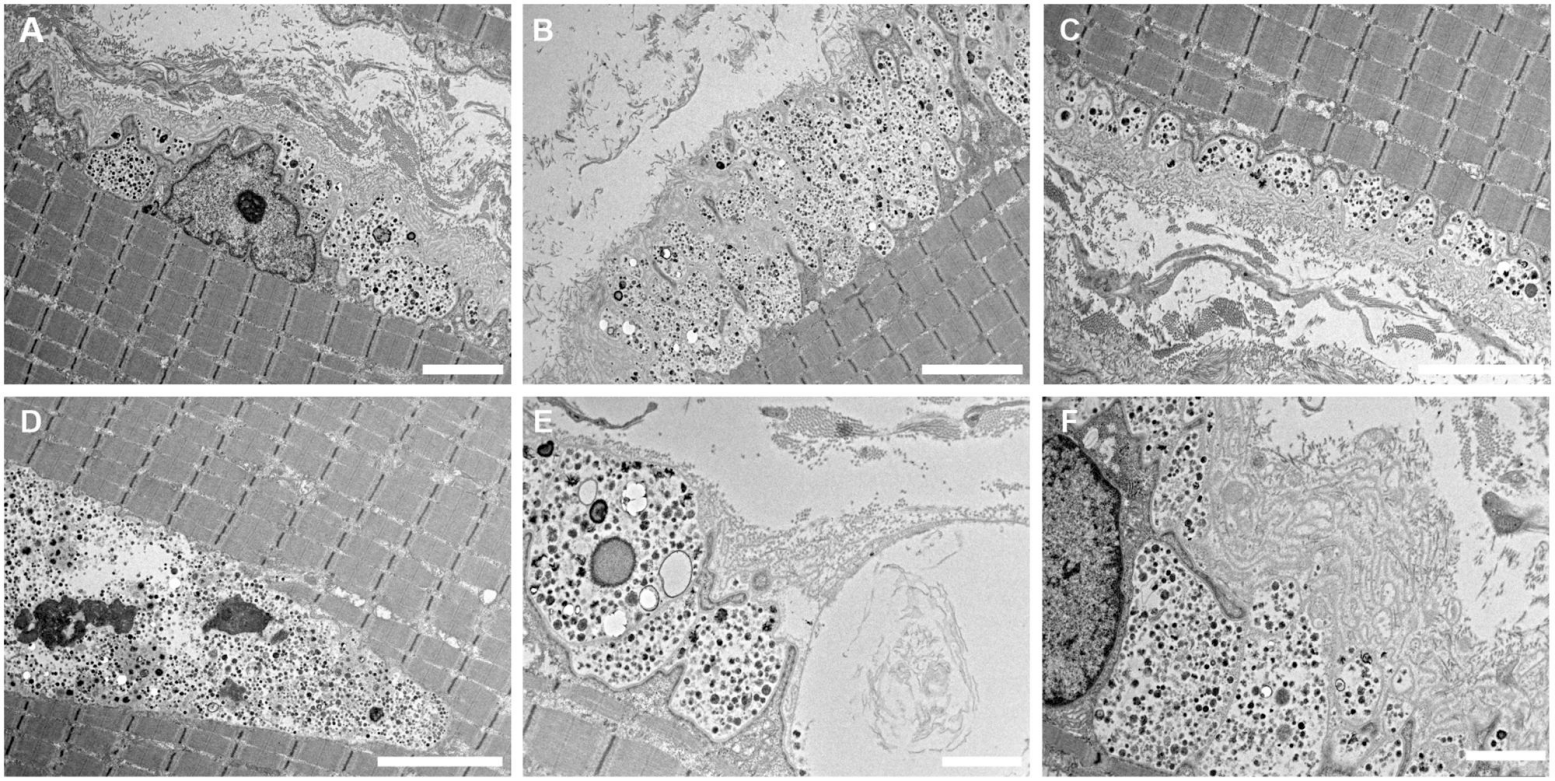
Ultrastructural features in muscle biopsy from a patient with XMEA. (A-F) Electron microscopy revealed numerous vacuoles located within the sarcolemma (A-F), between layers of the basal lamina (A, B, F), and in the cytoplasm (D). Vacuoles contained autophagic material, granular deposits, and mineralized structures likely corresponding to calcium accumulations (E). Replication of the basal lamina and accumulation of collagen within the extracellular matrix were also observed (A, C). Despite these abnormalities, sarcomere architecture appears well preserved. Scale bars: 10 μm (A, E); 5 μm (B, C, D); 2 μm (F). Images are from patient *c*.

Ultrastructural analysis was performed in six of the nine patients (*a* to *f*) by electron microscopy on available muscle samples. The ultrastructural features are summarized in Table 3 and shown in Figure 2. Overall, the ultrastructural characteristics of the muscle tissue were similar among patients. We confirmed the presence of numerous vacuoles in the sarcolemma and cytoplasm. These vacuoles contained autophagic material, which appeared granular. This material was also observed in most patients between the layers of the basal lamina or in the extracellular matrix, suggesting exocytosis of this material (Figure 2B, F). These structures occupy a large part of the muscle fiber surface (Figure 2D). Occasionally, they were associated with mineralized structures, likely corresponding to calcium accumulation (Figure 2E). In all muscle biopsy specimens, we detected a replication of the basal lamina on most of the abnormal muscle fibers and a significant accumulation of collagen in the extracellular matrix (Figure 2F). Occasionally, network disorganization was observed in patients *b* and *e*, nuclear clusters in the patient *e*, and paracrystallin inclusions in the mitochondria of patient *a*.

### Proteomic profiling

**Figure 3.**
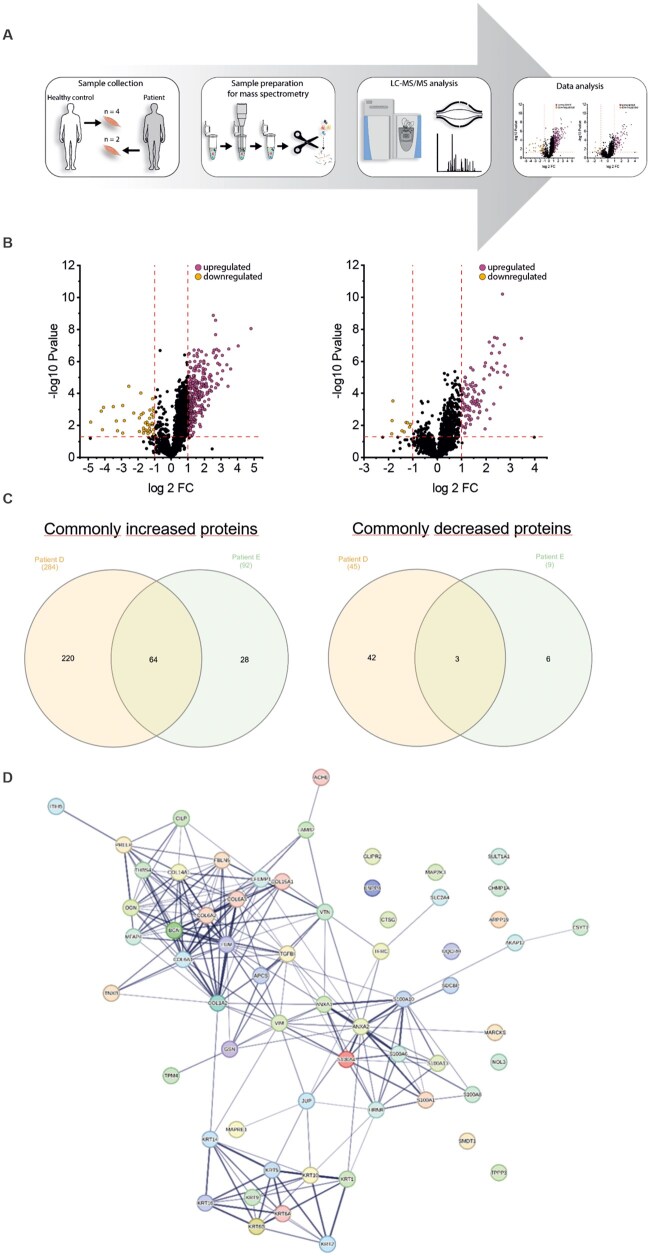
Results of proteomic profiling. (A) Schematic workflow of the applied mass spectrometry based analytical workflow. (B) Volcano plots illustrating dysregulation of muscle proteins in patient d (left panel) and patient e (right panel), respectively. (C) Venn diagrams illustrating proteins commonly increased (left panel) and decreased (right panel) in skeletal muscle of XMEA patients in terms of tissue markers. (D) String network analysis illustrating the functional interplay and interdependence of dysregulated proteins.

Proteomic profiling was conducted using muscle biopsy protein extracts from two patients (*d* and *e*) and matching healthy controls (Figure 3A). This approach enabled the robust quantification of 1637–1780 proteins, of which 101–329 presented with at least two unique peptides and a p-ANOVA < 0.05. Notably, the degree of myopathology correlated with the extent of protein dysregulations. The proteomic response in patient *d* (severe clinical severity, mutation c.163 + 4A>G) revealed an increase in the abundance of 284 proteins and a decrease in the abundance of 45 proteins. Proteomic analysis of patient *e* (mild clinical severity, mutation c.124A>G) revealed an increase in the abundance of 92 proteins and a decrease in 9 proteins. In total, 67 proteins were significantly dysregulated in both patients, with 64 proteins increased and 3 decreased (Figure 3B, C; Supplementary Tables 1, 2). This STRING network analysis reveals a dense cluster of interactions among extracellular matrix proteins (notably multiple collagen subunits such as COL1A2, COL6A1, COL6A2, COL6A3, and COL5A1) and cytoskeletal components (keratins, vimentin, actins). Central hub proteins like VIM, COL1A2, ANXA2, and S100A6 serve as key connectors between extracellular matrix, cytoskeletal, and regulatory proteins, highlighting their importance in structural integrity and signaling. The network suggests strong crosstalk between extracellular matrix organization, cytoskeletal dynamics, and calcium-binding proteins, which may play roles in processes such as cell adhesion, migration, and tissue remodeling (Figure 3D).

### Verification of proteomic data by immunofluorescence analyses

**Figure 4.**
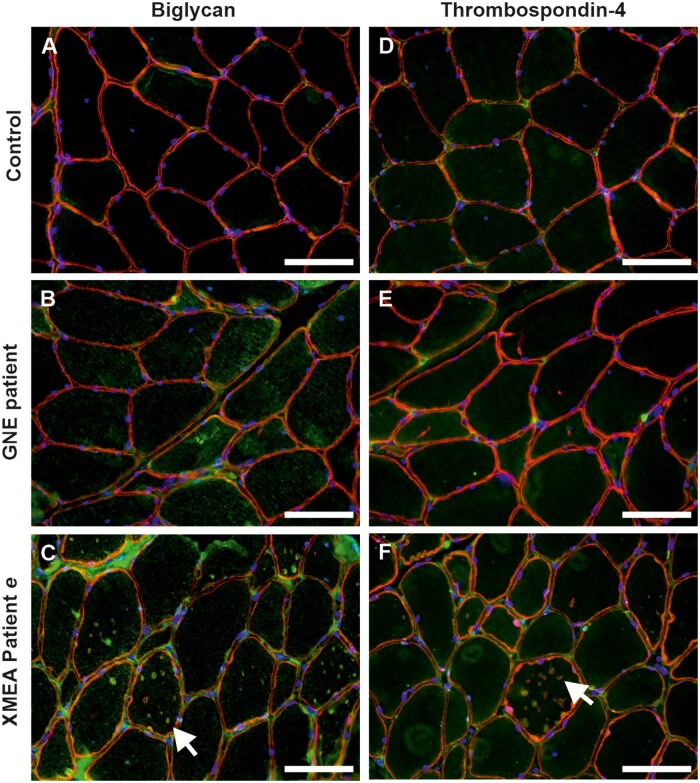
Preliminary validation of proteomic findings by immunofluorescence analysis. Immunofluorescence staining confirmed increased expression of thrombospondin-4 (THBS4) (left panel) and biglycan (right panel), identified by proteomic analysis, in the XMEA patient *e*. Muscle sections from a normal disease control (A and D), a patient with GNE myopathy (B and E) and the XMEA patient *e* (C and F) were analyzed. Co-staining with sarcoglycan (red) or laminin (green) was used to delineate the sarcolemma. Arrows indicate positive staining within cytoplasmic vacuoles. Nuclei were counterstained with DAPI (blue). Scale bars: 50 μm.

Biglycan and thrombospondin-4 (THBS4), two extracellular matrix proteins synthesized in the ER/Golgi and secreted via vesicular transport, identified through our proteomic discovery approach, were studied using immunofluorescence analyses. These antibodies were tested on muscle samples from the two selected patients (patient *e*, Figure 4 and patient *d*, Supplementary Figure 9), available in frozen form, and compared to one control subject and one GNE myopathy patient. In patient *e*, biglycan was increased in the extracellular matrix and cytoplasmic vacuoles (Figure 4C), while no significant labeling was observed in the control subject or the GNE myopathy patient (Figure 4A, B). Unfortunately, no muscle tissue was available for biglycan staining in patient *d*. THBS4 was slightly increased in the extracellular matrix and cytoplasmic vacuoles of patient *e* (Figure 4F), while no significant labeling was observed in the control subject or the GNE myopathy patient (Figure 4D, E). In patient *d*, THBS4 was increased in the extracellular matrix without significant labeling in the cytoplasmic vacuoles (Supplementary Figure 9).

## DISCUSSION

We report a large series of nine patients from six unrelated families carrying pathogenic *VMA21* variants, including two patients from the same family previously described.^20^ Through the integration of proteomic profiling and comprehensive histopathological analyses, we expand the phenotypic spectrum of XMEA and provide novel insights into its underlying pathophysiology. Importantly, our proteomic approach identified diagnostic-relevant protein signatures which are in line with muscle pathology, offering new potential biomarkers for disease stratification.

### From genotype-phenotype correlation to broader clinical spectrum

Although XMEA is classically considered a childhood-onset vacuolar myopathy, we report adult-onset disease in four out of nine patients. Three of these patients harbored the same intronic mutation upstream of exon 3 (c.164-7T>G), a variant frequently reported in the literature^3^^,^^8^ and associated with milder clinical courses and later disease onset. The fourth patient, presenting similarly mild symptoms, carried a novel variant in the 3’UTR (c.*124A>G). These findings emphasize that XMEA should be included in the differential diagnosis of adult-onset vacuolar myopathies and limb-girdle muscular dystrophies (LGMD), as recently advocated.^3^ Conversely, patients carrying the c.163 + 4A>G variant, the second most frequent mutation, displayed more severe disease, with one individual losing ambulation by 48 years of age (patient *d*), corroborating previous reports.^2^^,^^3^^,^^8^^,^^14^^,^^22^

Muscle weakness predominated in the pelvic and femoral girdles and extended variably to proximal and distal segments, but no patients exhibited ptosis, external ophthalmoplegia, facial weakness, or dysphagia, consistent with the classical phenotype.^12^ Cardiac involvement was observed in three patients, presenting as rhythm disturbances requiring pacemaker implantation, left anterior hemiblock, and aortic valve replacement. While a direct association with XMEA cannot be confirmed, these findings are consistent with emerging evidence that cardiac manifestations, though rare, may occur in this condition.^3^^,^^5^ In light of this, cardiac monitoring could be considered in the management of XMEA, particularly in severe cases.

### From histopathological characterization to molecular insights

Muscle biopsies revealed hallmark XMEA features: numerous cytoplasmic vacuoles (affecting 4–58% of fibers), high rates of internalized nuclei (4–34%), fiber splitting, increased endomysial connective tissue, and marked fiber size variability. Immunohistochemical analysis confirmed the autophagic nature of the vacuoles with positive staining for LC3, p62, and LAMP-2. The preserved LAMP-2 expression helped exclude Danon disease,^23^ and the absence of VCP or hnRNP aggregates ruled out multisystem proteinopathies.^24^ C5b-9 deposition along vacuolated fibers, a highly characteristic finding for XMEA,^20^^,^^25^ was consistently observed, although its pathogenic significance remains elusive. Cytoplasmic TDP-43 aggregates, recently reported in XMEA,^3^ were also noted. Electron microscopy further revealed classic ultrastructural abnormalities: abundant subsarcolemmal and cytoplasmic vacuoles, extensive basal lamina duplication, and significant collagen accumulation within the extracellular matrix.^12^

Proteomics offers an unbiased approach to uncover molecular perturbations in rare neuromuscular diseases.^26^ Here, comparative proteomic profiling in two patients with distinct histopathological severities (patients *d* and *e*) revealed dysregulation of proteins involved in complement activation, proteostasis, mitochondrial function, and cytoskeletal integrity. Our STRING network analysis of dysregulated proteins highlights strong interactions between extracellular matrix proteins (especially collagens) and cytoskeletal elements, with hub proteins like VIM, COL1A2, and ANXA2 linking these systems. The connectivity suggests coordinated dysregulation of structural integrity and fibrotic remodeling processes. These findings support the notion that autophagic vacuolization secondarily disrupts multiple cellular pathways beyond the protein clearance machinery. Importantly, the extent of proteomic alterations revealed a greater number of dysregulated proteins in the more severely affected muscle, highlighting, (based on data intersection and thus definition of commonly dysregulated proteins), potential biomarkers for disease stratification. Immunofluorescence validation focused on two extracellular matrix proteins, biglycan and THBS4, both synthesized in the ER/Golgi and secreted via vesicular transport and both already linked to myopathology (defined by advanced stage of myofibrillar breakdowns, lesions, presence of lysosomal vesicles accompanied by abundant autophagic vacuoles) in SMALED2, based on dominant *BICD2* variants.^27^ These proteins were upregulated in patient *e* but absent in GNE myopathy and healthy controls. Furthermore, THBS4 expression was less prominent in the clinically milder patient (patient *e*) compared to patient *d*, reinforcing the correlation between protein dysregulation and pathological severity and by the same token introduces THBS4 as a tissue marker for disease activity. It is important to note that the proteomics experiments were performed on only two patients, one of whom carries a novel mutation that is unique to their case (patient *e*), while the other has a known mutation (patient *d*). This limited sample size precludes direct comparison with patients harboring more common mutations. The choice to focus on these specific patients was driven by the availability of tissue samples, and this limitation should be considered when interpreting the findings. Future studies including a broader cohort of patients with more common/shared mutations would provide a more comprehensive understanding of the proteomic landscape in XMEA.

These findings, although preliminary, together identify novel candidate markers for XMEA and emphasize the diagnostic and pathophysiological relevance of proteomic profiling. They further suggest that vesicular transport disturbances may contribute to disease pathogenesis beyond lysosomal dysfunction, an important aspect in the definition of diverse starting points for therapeutic intervention concepts.

## CONCLUSIONS

Through this large French cohort of genetically confirmed XMEA patients, this study broadens the clinical and pathological understanding of VMA21-related myopathy. We present a new genotype and demonstrate that XMEA can manifest beyond childhood-onset, including adult-onset forms, although this remains to be confirmed by long-term follow-up studies. The integration of histopathological, ultrastructural, and proteomic analyses strengthens the link between specific molecular perturbations and disease severity. By identifying disease-associated proteomic signatures, we provide novel insights into XMEA pathogenesis and suggest potential tissue biomarkers for improved diagnostic stratification. These findings emphasize the value of combining systematic clinicopathological evaluation with molecular approaches to refine the diagnosis of vacuolar myopathies.

## ETHICS APPROVAL AND CONSENT TO PARTICIPATE

This study is a retrospective analysis of clinical and morphological data. All biopsies were done in a diagnostic context and written informed consent was obtained from the patients at the time of the biopsy. All the procedures were performed in compliance with the ethical standards of the 1975 Declaration of Helsinki, revised in 2013.

## Supplementary Material

nlaf134_Supplementary_Data

## Data Availability

All data generated or analyzed during this study are included in this published article [and its supplementary information files].
