# Supplementary material for: Clinical, morphological, and molecular characterization of patients with X-linked myopathy with excessive autophagy (XMEA)
Source: J Neuropathol Exp Neurol. 2025 Nov 27;85(4):351–62. doi: 10.1093/jnen/nlaf134 (PMC13017771; doi:10.1093/jnen/nlaf134)
Supplement: nlaf134_Supplementary_Data [file nlaf134_supplementary_data.zip › Supplementary Tables 1 and 2.docx]

**Supplementary Table1: Summary of significant up-regulated proteins in Patient d and Patient e.**

| **Accession** | **Gene** | **Description** | **Name** | **Unique Peptides** | **Patient d /Ctrl** | **Patient e /Ctrl** |
| --- | --- | --- | --- | --- | --- | --- |
| P04259 | KRT6B | Keratin, type II cytoskeletal 6B | K2C6B | 2 | 4.01 | 10.97 |
| O75339 | CILP | Cartilage intermediate layer protein 1 | CILP1 | 7 | 5.85 | 6.95 |
| Q9BW30 | TPPP3 | Tubulin polymerization-promoting protein family member 3 | TPPP3 | 3 | 7.42 | 6.83 |
| P08311 | CTSG | Cathepsin G | CATG | 2 | 3.88 | 6.73 |
| P13645 | KRT10 | Keratin, type I cytoskeletal 10 | K1C10 | 27 | 6.43 | 6.46 |
| P26447 | S100A4 | Protein S100-A4 | S10A4 | 4 | 6.40 | 6.08 |
| P04004 | VTN | Vitronectin | VTNC | 7 | 6.36 | 5.90 |
| P08779 | KRT16 | Keratin, type I cytoskeletal 16 | K1C16 | 9 | 2.18 | 5.59 |
| P35908 | KRT2 | Keratin, type II cytoskeletal 2 epidermal | K22E | 22 | 5.83 | 5.57 |
| Q99584 | S100A13 | Protein S100-A13 | S10AD | 3 | 7.64 | 5.50 |
| P35527 | KRT9 | Keratin, type I cytoskeletal 9 | K1C9 | 27 | 3.19 | 5.13 |
| P35443 | THBS4 | Thrombospondin-4 | TSP4 | 6 | 10.26 | 4.96 |
| P04264 | KRT1 | Keratin, type II cytoskeletal 1 | K2C1 | 33 | 3.58 | 4.91 |
| P13647 | KRT5 | Keratin, type II cytoskeletal 5 | K2C5 | 8 | 3.79 | 4.70 |
| Q86YZ3 | HRNR | Hornerin | HORN | 4 | 2.52 | 4.70 |
| P51888 | PRELP | Prolargin | PRELP | 11 | 3.74 | 4.49 |
| P02538 | KRT6A | Keratin, type II cytoskeletal 6A | K2C6A | 22 | 2.75 | 4.43 |
| Q02952 | AKAP12 | A-kinase anchor protein 12 | AKA12 | 2 | 2.65 | 4.10 |
| P06703 | S100A6 | Protein S100-A6 | S10A6 | 3 | 6.66 | 3.95 |
| P02533 | KRT14 | Keratin, type I cytoskeletal 14 | K1C14 | 18 | 3.10 | 3.87 |
| P20774 | OGN | Mimecan | MIME | 12 | 2.51 | 3.82 |
| Q9UBX5 | FBLN5 | Fibulin-5 | FBLN5 | 4 | 8.55 | 3.79 |
| P05109 | S100A8 | Protein S100-A8 | S10A8 | 2 | 2.27 | 3.51 |
| P02743 | APCS | Serum amyloid P-component | SAMP | 6 | 6.78 | 3.38 |
| P51884 | LUM | Lumican | LUM | 12 | 2.49 | 3.28 |
| P06396 | GSN | Gelsolin | GELS | 26 | 3.90 | 3.28 |
| P22303 | ACHE | Acetylcholinesterase | ACES | 2 | 3.48 | 3.22 |
| P12111 | COL6A3 | Collagen alpha-3(VI) chain | CO6A3 | 94 | 3.72 | 3.13 |
| P08123 | COL1A2 | Collagen alpha-2(I) chain | CO1A2 | 11 | 2.04 | 3.11 |
| P67936 | TPM4 | Tropomyosin alpha-4 chain | TPM4 | 3 | 3.33 | 3.03 |
| Q9H4I9 | SMDT1 | Essential MCU regulator, mitochondrial | EMRE | 2 | 2.70 | 2.96 |
| Q9Y6X5 | ENPP4 | Bis(5'-adenosyl)-triphosphatase ENPP4 | ENPP4 | 2 | 2.50 | 2.95 |
| P55083 | MFAP4 | Microfibril-associated glycoprotein 4 | MFAP4 | 3 | 12.09 | 2.95 |
| P60903 | S100A10 | Protein S100-A10 | S10AA | 2 | 6.05 | 2.94 |
| Q15691 | MAPRE1 | Microtubule-associated protein RP/EB family member 1 | MARE1 | 2 | 2.93 | 2.86 |
| P14923 | JUP | Junction plakoglobin | PLAK | 5 | 2.25 | 2.85 |
| P02786 | TFRC | Transferrin receptor protein 1 | TFR1 | 3 | 2.68 | 2.78 |
| Q05707 | COL14A1 | Collagen alpha-1(XIV) chain | COEA1 | 3 | 2.00 | 2.77 |
| P29966 | MARCKS | Myristoylated alanine-rich C-kinase substrate | MARCS | 3 | 4.46 | 2.75 |
| P23297 | S100A1 | Protein S100-A1 | S10A1 | 6 | 5.45 | 2.68 |
| Q9BSJ8 | ESYT1 | Extended synaptotagmin-1 | ESYT1 | 16 | 2.52 | 2.60 |
| Q9H4G4 | GLIPR2 | Golgi-associated plant pathogenesis-related protein 1 | GAPR1 | 2 | 4.07 | 2.55 |
| P12110 | COL6A2 | Collagen alpha-2(VI) chain | CO6A2 | 22 | 3.34 | 2.55 |
| Q12805 | EFEMP1 | EGF-containing fibulin-like extracellular matrix protein 1 | FBLN3 | 2 | 7.03 | 2.54 |
| P12109 | COL6A1 | Collagen alpha-1(VI) chain | CO6A1 | 26 | 3.20 | 2.53 |
| Q86UX2 | ITIH5 | Inter-alpha-trypsin inhibitor heavy chain H5 | ITIH5 | 6 | 2.82 | 2.46 |
| P04083 | ANXA1 | Annexin A1 | ANXA1 | 14 | 4.35 | 2.42 |
| P14672 | SLC2A4 | Solute carrier family 2, facilitated glucose transporter member 4 | GTR4 | 4 | 2.19 | 2.41 |
| O60936 | NOL3 | Nucleolar protein 3 | NOL3 | 3 | 3.00 | 2.38 |
| Q15582 | TGFBI | Transforming growth factor-beta-induced protein ig-h3 | BGH3 | 12 | 2.92 | 2.34 |
| P07355 | ANXA2 | Annexin A2 | ANXA2 | 26 | 3.38 | 2.30 |
| O00560 | SDCBP | Syntenin-1 | SDCB1 | 3 | 2.21 | 2.26 |
| P22105 | TNXB | Tenascin-X | TENX | 23 | 2.21 | 2.24 |
| P39059 | COL15A1 | Collagen alpha-1(XV) chain | COFA1 | 12 | 3.22 | 2.23 |
| P55268 | LAMB2 | Laminin subunit beta-2 | LAMB2 | 39 | 2.77 | 2.21 |
| P21810 | BGN | Biglycan | PGS1 | 6 | 3.68 | 2.20 |
| P07919 | UQCRH | Cytochrome b-c1 complex subunit 6, mitochondrial | QCR6 | 2 | 3.02 | 2.17 |
| P50225 | SULT1A1 | Sulfotransferase 1A1 | ST1A1 | 3 | 2.27 | 2.15 |
| P08670 | VIM | Vimentin | VIME | 38 | 3.96 | 2.14 |
| P01859 | IGHG2 | Immunoglobulin heavy constant gamma 2 | IGHG2 | 6 | 4.51 | 2.12 |
| P56211 | ARPP19 | cAMP-regulated phosphoprotein 19 | ARP19 | 2 | 2.15 | 2.12 |
| Q9HD42 | CHMP1A | Charged multivesicular body protein 1a | CHM1A | 2 | 2.30 | 2.03 |
| A0A0B4J1X5 | IGHV3-74 | Immunoglobulin heavy variable 3-74 | HV374 | 2 | 2.64 | 2.03 |
| P46734 | MAP2K3 | Dual specificity mitogen-activated protein kinase kinase 3 | MP2K3 | 7 | 2.11 | 2.03 |

**Supplementary Table 2: Summary of significant down-regulated proteins in Patient d and Patient e.**

| **Accession** | **Gene** | **Description** | **Name** | **Unique Peptides** | **Patient d /Ctrl** | **Patient e /Ctrl** |
| --- | --- | --- | --- | --- | --- | --- |
| P00395 | MT-CO1 | Cytochrome c oxidase subunit 1 | COX1 | 4 | 0.35 | 0.37 |
| P14854 | COX6B1 | Cytochrome c oxidase subunit 6B1 | CX6B1 | 5 | 0.32 | 0.39 |
| Q96DG6 | CMBL | Carboxymethylenebutenolidase homolog | CMBL | 14 | 0.21 | 0.42 |
