# Supplementary material for: Clinical, morphological, and molecular characterization of patients with X-linked myopathy with excessive autophagy (XMEA)
Source: J Neuropathol Exp Neurol. 2025 Nov 27;85(4):351–62. doi: 10.1093/jnen/nlaf134 (PMC13017771; doi:10.1093/jnen/nlaf134)
Supplement: nlaf134_Supplementary_Data [file nlaf134_supplementary_data.zip › Rays redone Merlet Supplementary Figure 2.pptx]

## Slide 1
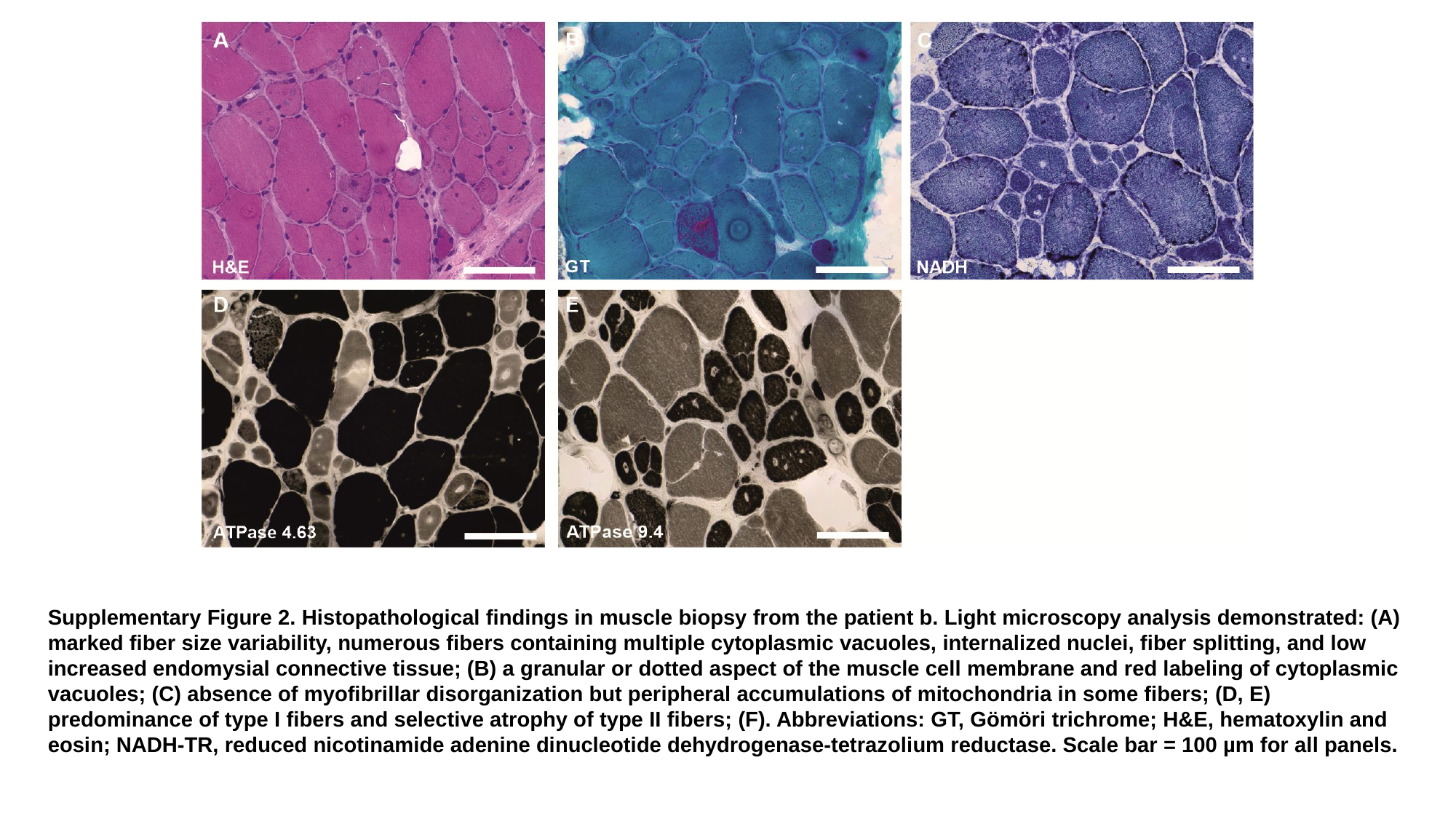

Supplementary Figure 2. Histopathological findings in muscle biopsy from the patient b. Light microscopy analysis demonstrated: (A) marked fiber size variability, numerous fibers containing multiple cytoplasmic vacuoles, internalized nuclei, fiber splitting, and low increased endomysial connective tissue; (B) a granular or dotted aspect of the muscle cell membrane and red labeling of cytoplasmic vacuoles; (C) absence of myofibrillar disorganization but peripheral accumulations of mitochondria in some fibers; (D, E) predominance of type I fibers and selective atrophy of type II fibers; (F). Abbreviations: GT, Gömöri trichrome; H&E, hematoxylin and eosin; NADH-TR, reduced nicotinamide adenine dinucleotide dehydrogenase-tetrazolium reductase. Scale bar = 100 µm for all panels.
