# Supplementary material for: Clinical, morphological, and molecular characterization of patients with X-linked myopathy with excessive autophagy (XMEA)
Source: J Neuropathol Exp Neurol. 2025 Nov 27;85(4):351–62. doi: 10.1093/jnen/nlaf134 (PMC13017771; doi:10.1093/jnen/nlaf134)
Supplement: nlaf134_Supplementary_Data [file nlaf134_supplementary_data.zip › Rays redone Merlet Supplementary Figure 4.pptx]

## Slide 1
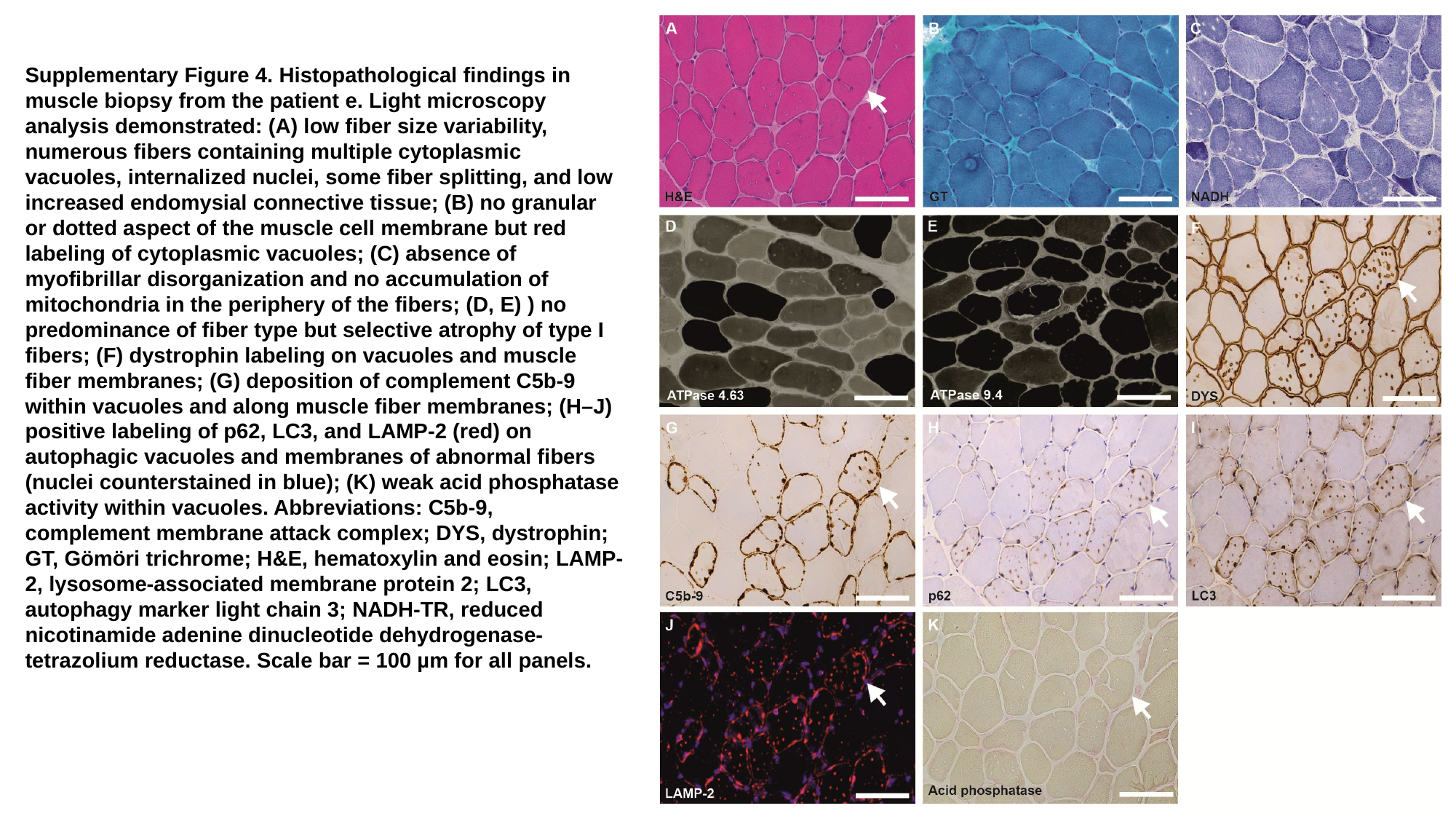

Supplementary Figure 4. Histopathological findings in muscle biopsy from the patient e. Light microscopy analysis demonstrated: (A) low fiber size variability, numerous fibers containing multiple cytoplasmic vacuoles, internalized nuclei, some fiber splitting, and low increased endomysial connective tissue; (B) no granular or dotted aspect of the muscle cell membrane but red labeling of cytoplasmic vacuoles; (C) absence of myofibrillar disorganization and no accumulation of mitochondria in the periphery of the fibers; (D, E) ) no predominance of fiber type but selective atrophy of type I fibers; (F) dystrophin labeling on vacuoles and muscle fiber membranes; (G) deposition of complement C5b-9 within vacuoles and along muscle fiber membranes; (H–J) positive labeling of p62, LC3, and LAMP-2 (red) on autophagic vacuoles and membranes of abnormal fibers (nuclei counterstained in blue); (K) weak acid phosphatase activity within vacuoles. Abbreviations: C5b-9, complement membrane attack complex; DYS, dystrophin; GT, Gömöri trichrome; H&E, hematoxylin and eosin; LAMP-2, lysosome-associated membrane protein 2; LC3, autophagy marker light chain 3; NADH-TR, reduced nicotinamide adenine dinucleotide dehydrogenase-tetrazolium reductase. Scale bar = 100 µm for all panels.
