# Supplementary material for: Clinical, morphological, and molecular characterization of patients with X-linked myopathy with excessive autophagy (XMEA)
Source: J Neuropathol Exp Neurol. 2025 Nov 27;85(4):351–62. doi: 10.1093/jnen/nlaf134 (PMC13017771; doi:10.1093/jnen/nlaf134)
Supplement: nlaf134_Supplementary_Data [file nlaf134_supplementary_data.zip › Rays redone Merlet Supplementary Figure 8.pptx]

## Slide 1
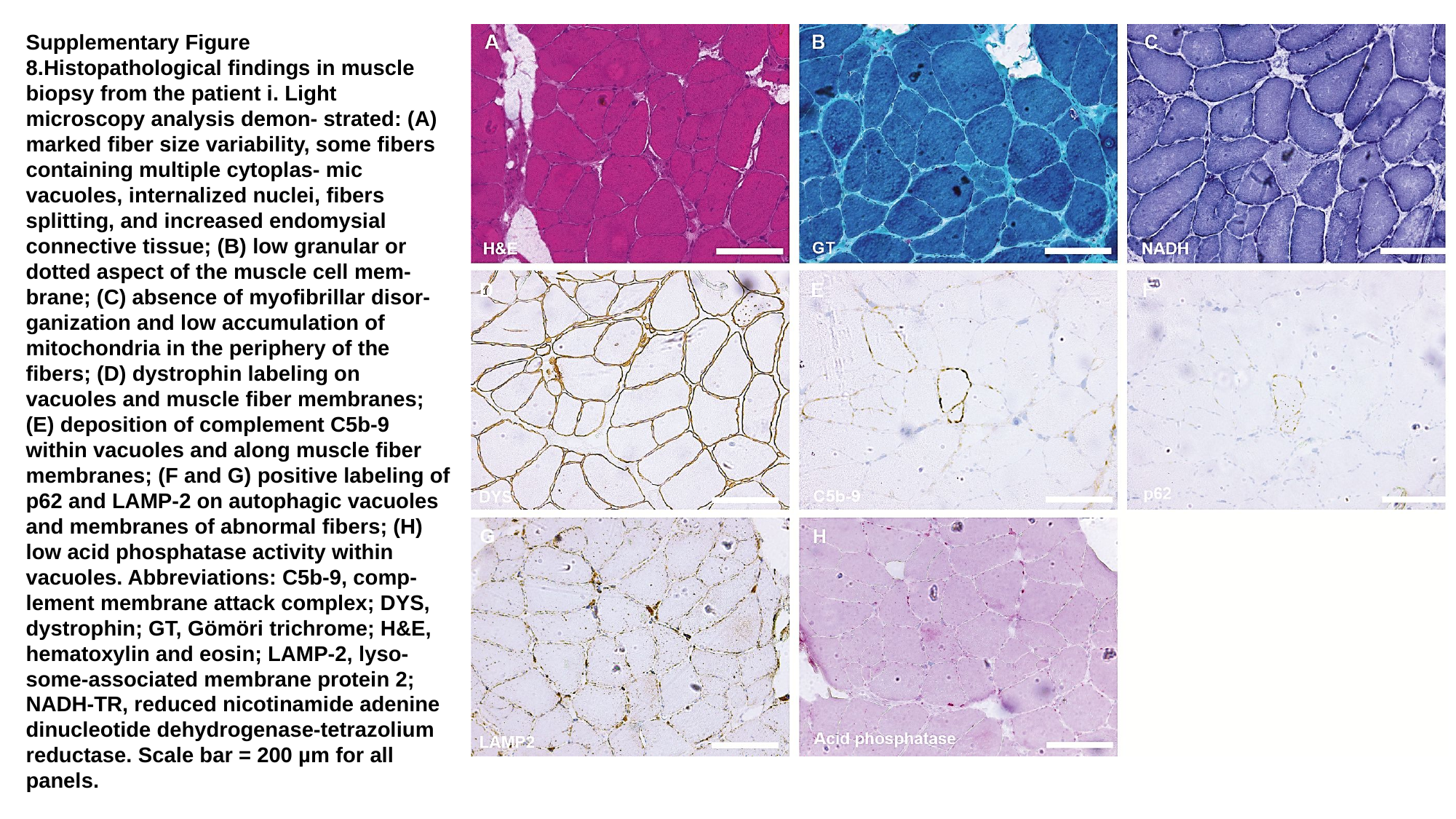

Supplementary Figure 8.Histopathological findings in muscle biopsy from the patient i. Light microscopy analysis demon- strated: (A) marked fiber size variability, some fibers containing multiple cytoplas- mic vacuoles, internalized nuclei, fibers splitting, and increased endomysial connective tissue; (B) low granular or dotted aspect of the muscle cell mem- brane; (C) absence of myofibrillar disor- ganization and low accumulation of mitochondria in the periphery of the fibers; (D) dystrophin labeling on vacuoles and muscle fiber membranes; (E) deposition of complement C5b-9 within vacuoles and along muscle fiber membranes; (F and G) positive labeling of p62 and LAMP-2 on autophagic vacuoles and membranes of abnormal fibers; (H) low acid phosphatase activity within vacuoles. Abbreviations: C5b-9, comp- lement membrane attack complex; DYS, dystrophin; GT, Gömöri trichrome; H&E, hematoxylin and eosin; LAMP-2, lyso- some-associated membrane protein 2; NADH-TR, reduced nicotinamide adenine dinucleotide dehydrogenase-tetrazolium reductase. Scale bar = 200 µm for all panels.
