# Supplementary material for: Clinical, morphological, and molecular characterization of patients with X-linked myopathy with excessive autophagy (XMEA)
Source: J Neuropathol Exp Neurol. 2025 Nov 27;85(4):351–62. doi: 10.1093/jnen/nlaf134 (PMC13017771; doi:10.1093/jnen/nlaf134)
Supplement: nlaf134_Supplementary_Data [file nlaf134_supplementary_data.zip › Rays redone Merlet Supplementary Figure 9.pptx]

## Slide 1
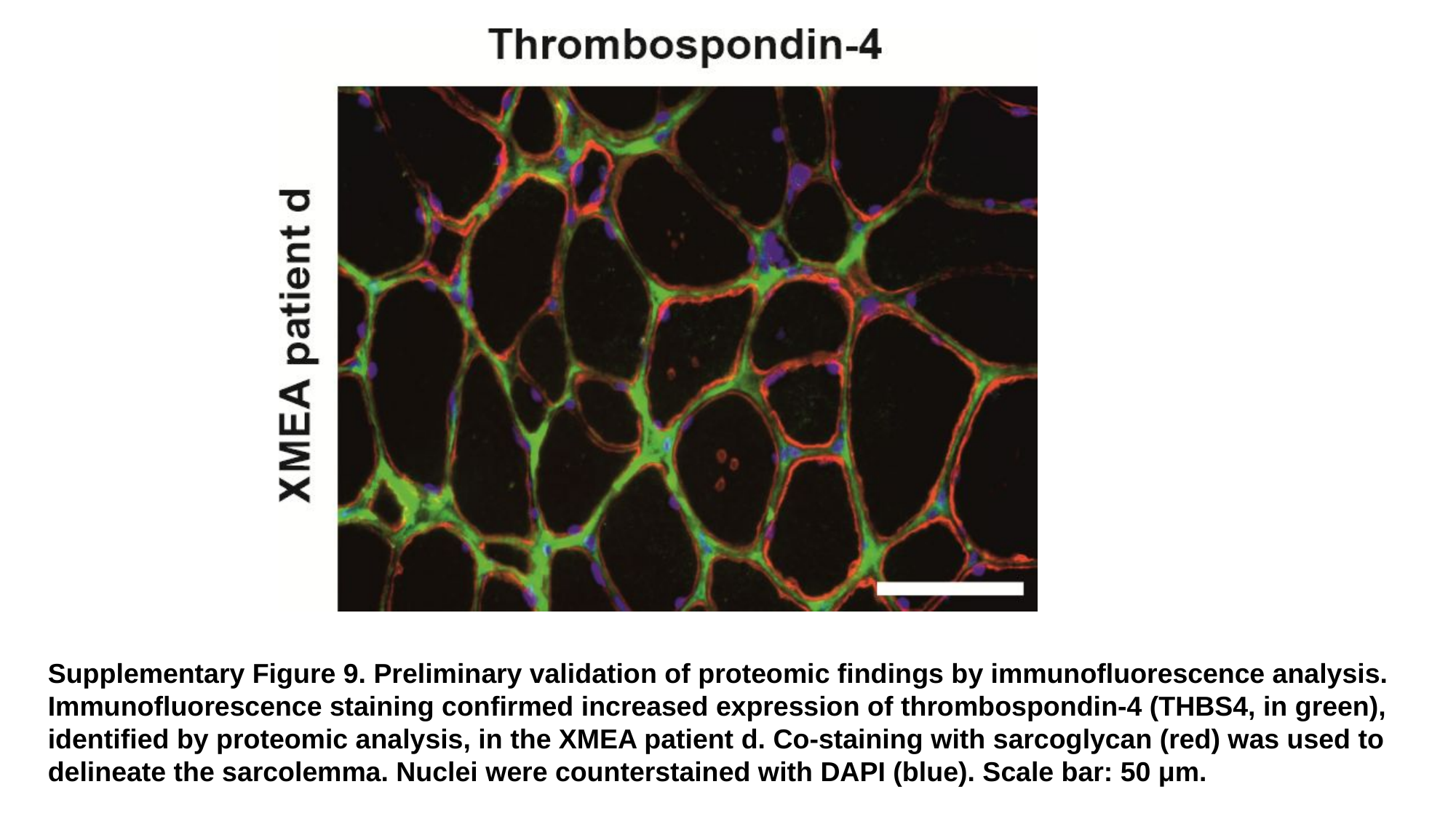

Supplementary Figure 9. Preliminary validation of proteomic findings by immunofluorescence analysis. Immunofluorescence staining confirmed increased expression of thrombospondin-4 (THBS4, in green), identified by proteomic analysis, in the XMEA patient d. Co-staining with sarcoglycan (red) was used to delineate the sarcolemma. Nuclei were counterstained with DAPI (blue). Scale bar: 50 μm.
